# Supplementary material for: Aging, inflammation and DNA damage in the somatic testicular niche with idiopathic germ cell aplasia
Source: Nat Commun. 2021 Sep 1;12:5205. doi: 10.1038/s41467-021-25544-0 (PMC8410861; doi:10.1038/s41467-021-25544-0)
Supplement: Supplementary file 1 — Supplementary Information [file 41467_2021_25544_MOESM1_ESM.pdf]

**SUPPLEMENTARY INFORMATION for the manuscript "Aging, inflammation and DNA damage in the somatic testicular niche with idiopathic germ cell aplasia"**

Massimo Alfano, Anna Sofia Tascini, Filippo Pederzoli, Irene Locatelli, Manuela Nebuloni, Francesca Giannese, Jose Manuel Garcia-Manteiga, Giovanni Tonon, Giada Amodio, Silvia Gregori, Alessandra Agresti, Francesco Montorsi, and Andrea Salonia

The Supplementary Information of this study consist of:

- 13 Supplementary Figures

- 36 Supplementary Datasets

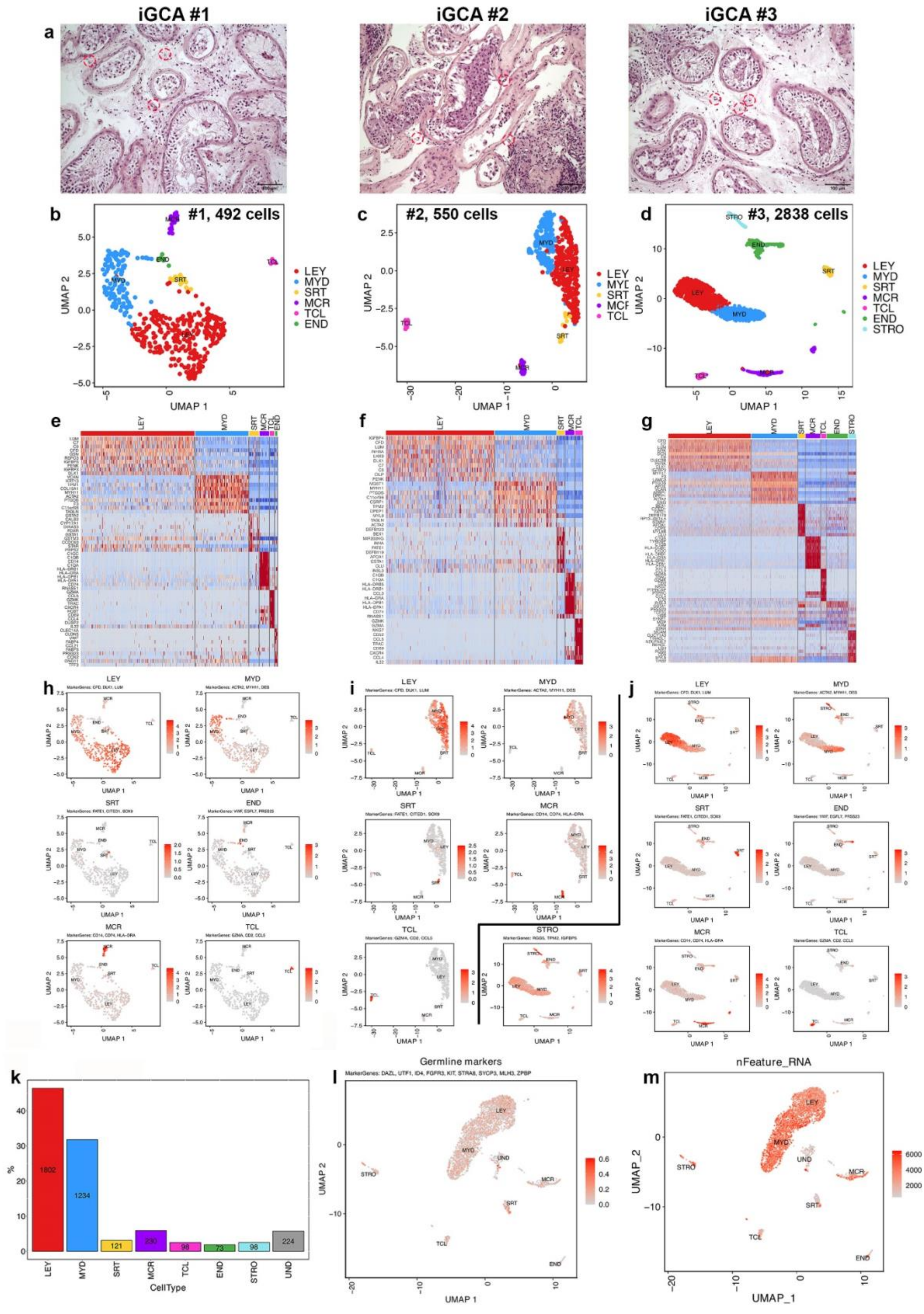


**Fig. S1. Clustering of somatic cells from 3 testes with iGCA through the analysis of marker genes expression. a** Hematoxylin-eosin staining of testis parenchyma from the three

iNOA specimens used in this study, showing the absence of germ cells, hyalinization of the basal membrane of the seminiferous tubules and presence of vessels (indicated by red dashed circles). **b, c, d** UMAP plot representing identity attribution for testis cells from tissue samples of 3 independent iNOA men with complete GCA, after removal of low quality cells as reported in the methods section. **e, f, g** Heatmaps showing the expression signature of the top 10 expressed genes in each cell type for each of the 3 donors. **h, i, j** UMAP plots showing the expression patterns of selected marker genes used to identify the different cell types for each of the 3 donors; red color indicates high expression, while light-grey low or no expression. **k** Relative abundance of the somatic cells in the 3 testes of iNOA men. **l** UMAP plot of the cells isolated from 3 testes with iGCA and based on germ line gene markers. **m** Number of RNAs features for each somatic cell population from the 3 testes with iGCA.

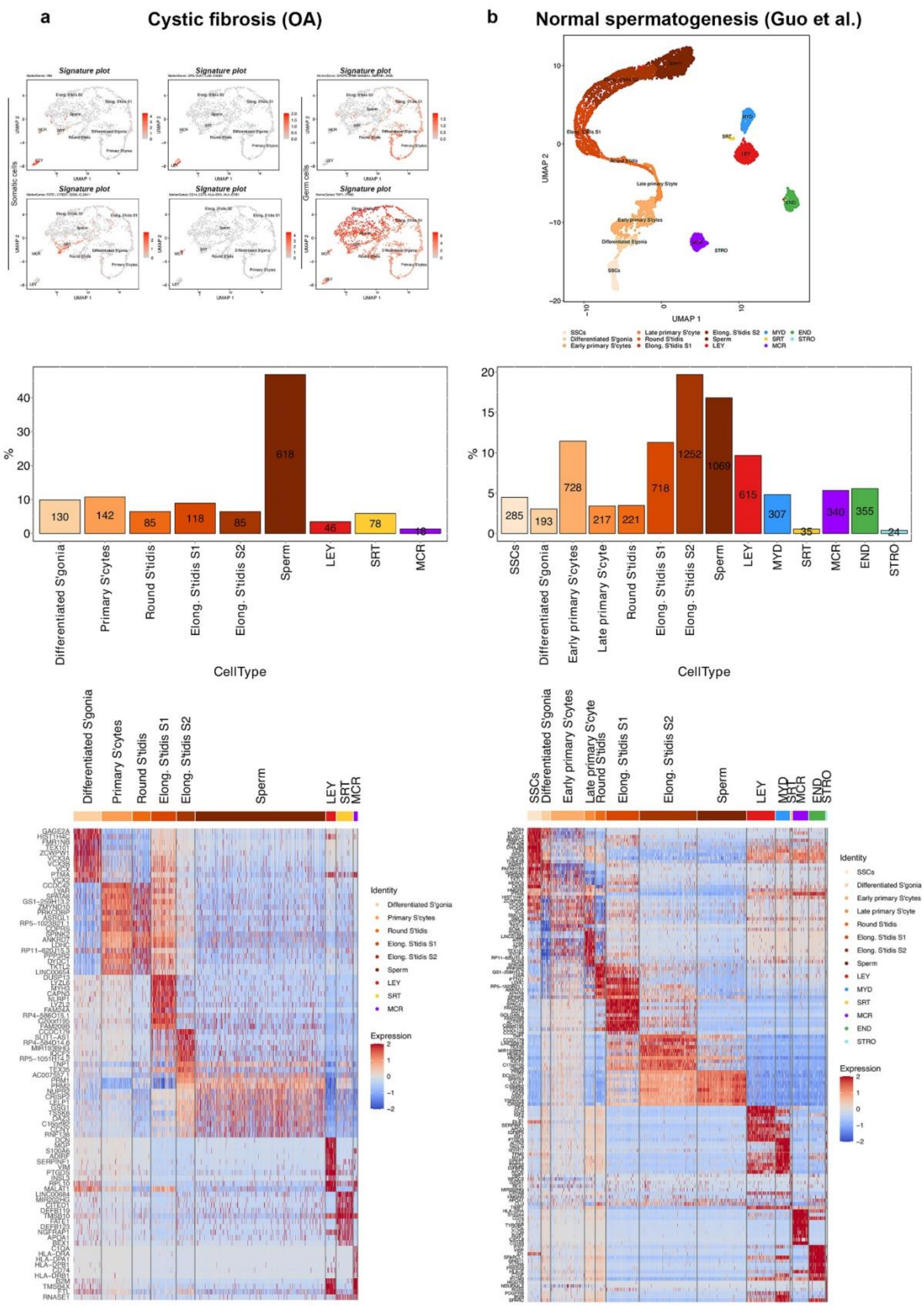


**Fig. S2. Cell partitioning from testis with obstructive azoospermia and normal spermatogenesis. a** UMAP plots showing the expression patterns of selected marker genes

used to identify somatic cells and germ cells in the testis of 1 man with obstructive azoospermia caused by mutation of *CFTR* gene (Supplementary information, Dataset S0), and (below) the relative abundance of cell populations based on gene markers shown in the heatmap. **b** UMAP and cluster analysis of data from normal spermatogenesis of three donors available from Guo et al.[15] (Supplementary information, Dataset S10), and (below) the relative abundance of cell populations based on gene markers shown in the heatmap.

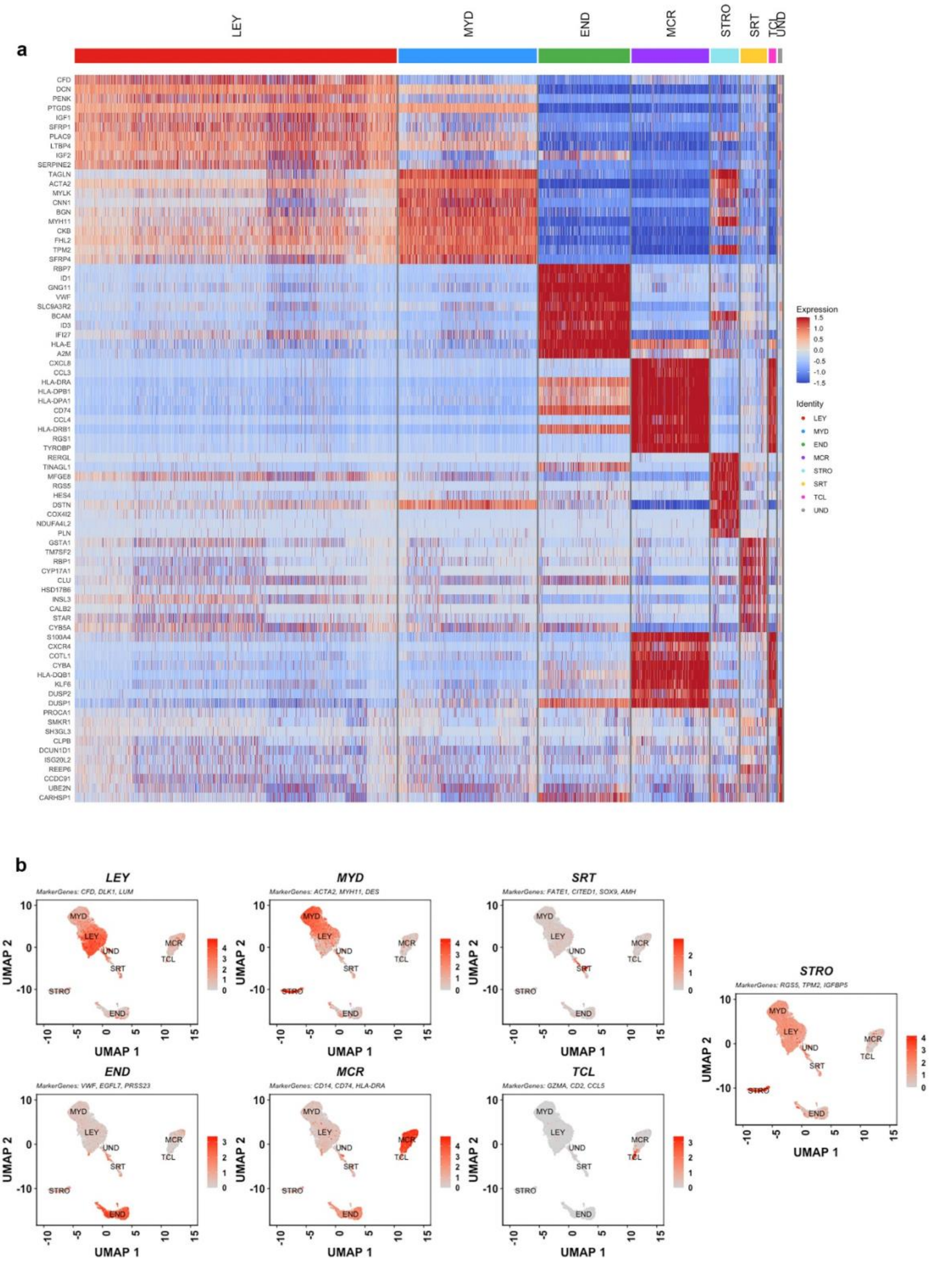


**Fig. S3. Integrated analysis of somatic cells from the testis with iGCA and normal spermatogenesis. a** Heatmap of marker genes used to identify each somatic cell populations from the testis with GCA (iNOA men, n=3) and normal spermatogenesis (Guo et al., n=3 and

Sohni et al, n = 2). **b** Visualization of all somatic cell populations based on gene markers from 3 testes with GCA (iNOA men) and 5 testes with normal spermatogenesis (from Guo et al. and Sohni et al.).

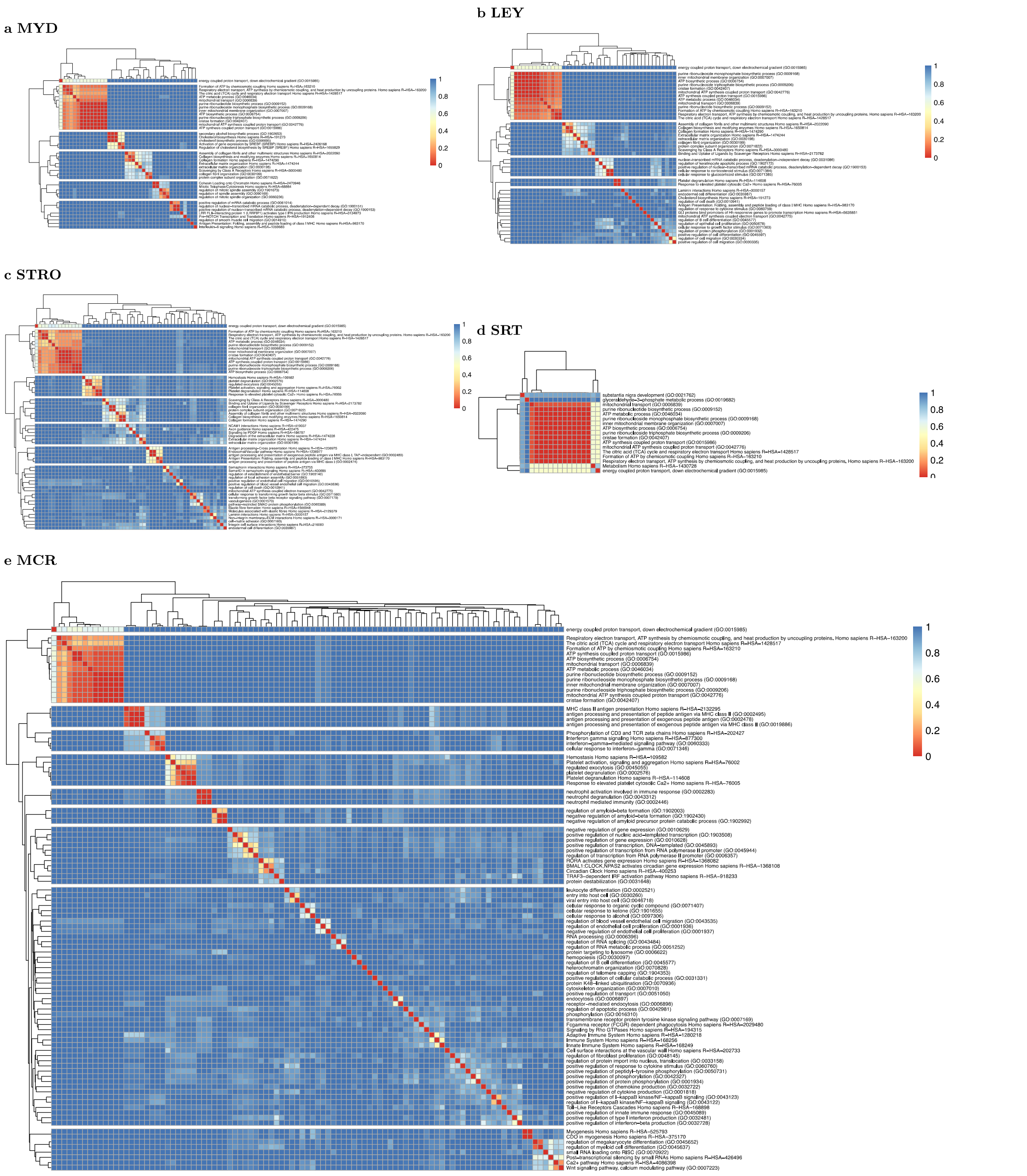


**Fig. S4. Upregulated pathways in somatic cells from testes with iGCA.** The Jaccard distance heatmaps with the CRAN package *philentrophy* was used to plot all significant (adjusted *P* value <0.05) pathways in the somatic cells from the testis with iGCA and identified from the Reactome_2016 dataset and GO biological process.

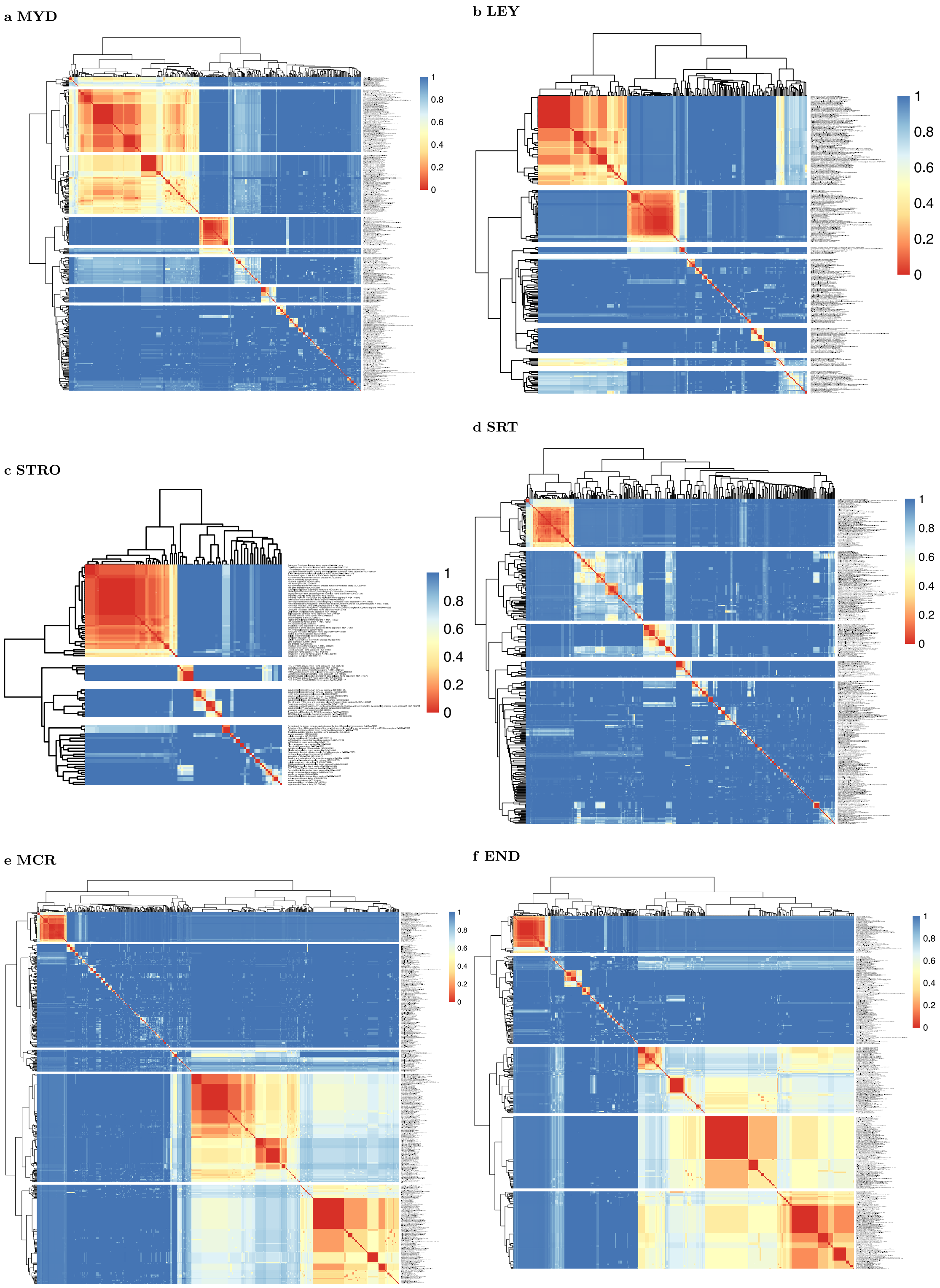


**Fig. S5. Downregulated pathways in somatic cells from the testes with iGCA.** The Jaccard distance heatmaps with the CRAN package *philentropy* was used to plot all significant (adjusted *P* value <0.05) pathways in the somatic cells from the testis with iGCA and identified from the Reactome_2016 dataset and GO biological process.

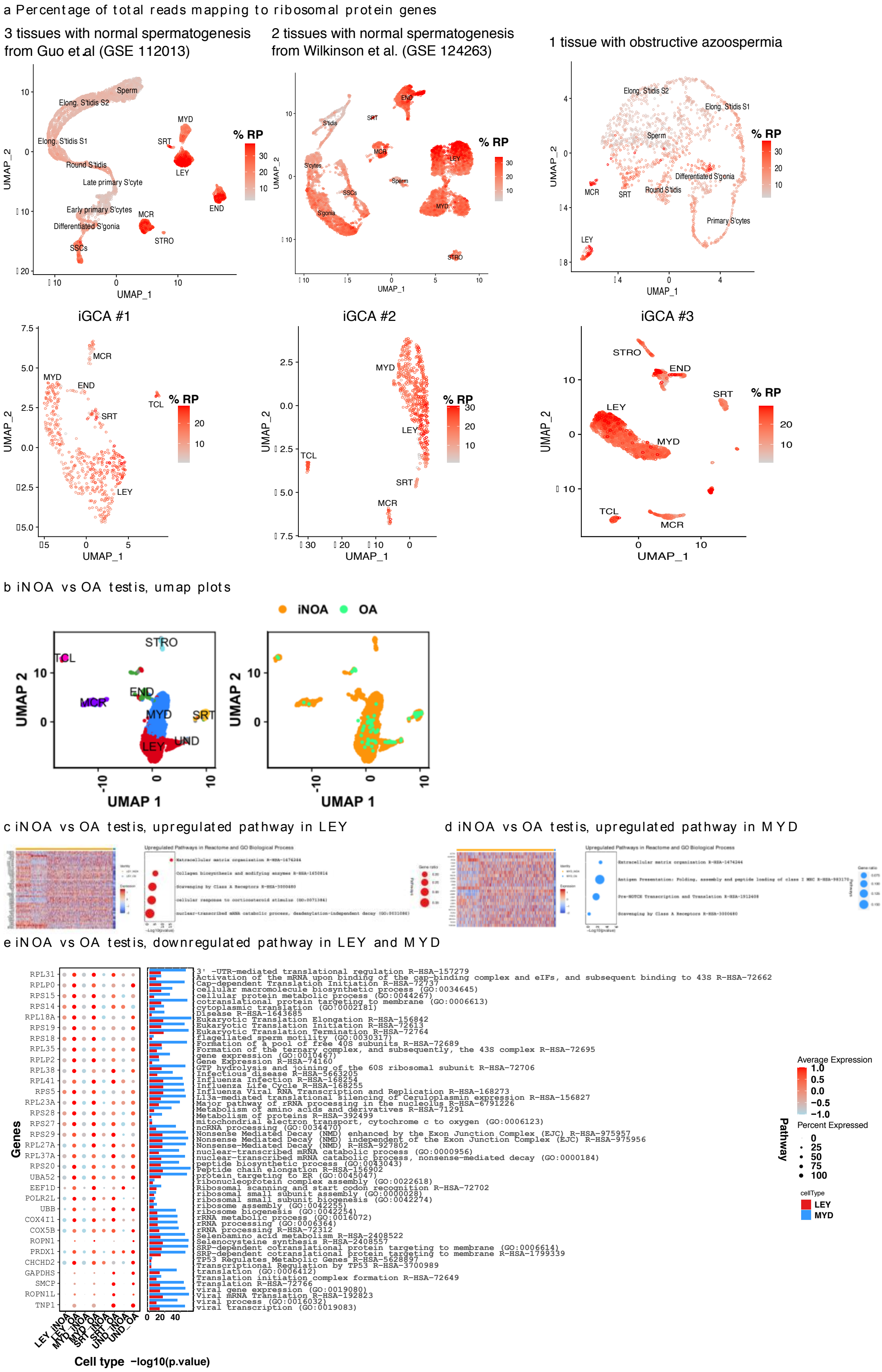

a Percentage of total reads mapping to ribosomal protein genes
3 tissues with normal spermatogenesis from Guo et al (GSE 112013)
2 tissues with normal spermatogenesis from Wilkinson et al. (GSE 124263)
1 tissue with obstructive azoospermia
% RP
UMAP_1
UMAP_2
Sperm
Elong. S'tidis S2
Elong. S'tidis S1
Round S'tidis
Late primary S'cyte
Early primary S'cytes
Differentiated S'gonia
SSCs
MYD
SRT
LEY
END
MCR
STRO
S'tids
S'cytes
Sgonia
Primary S'cytes
iGCA #1
iGCA #2
iGCA #3
TCL
b iNOA vs OA testis, umap plots
iNOA
OA
UMAP 1
UMAP 2
UND
c iNOA vs OA testis, upregulated pathway in LEY
d iNOA vs OA testis, upregulated pathway in MYD
e iNOA vs OA testis, downregulated pathway in LEY and MYD
RPL31
RPLP0
RPS15
RPS14
RPL18A
RPS19
RPS18
RPL35
RPLP2
RPL38
RPL41
RPS5
RPL23A
RPS28
RPS27
RPS29
RPL27A
RPL37A
RPS20
UBA52
EEF1D
POLR2L
UBB
COX411
COX5B
ROPN1
PRDX1
CHCHD2
GAPDHS
SMCP
ROPN1L
TNP1
Genes
LEY_iNOA
LEY_OA
MYD_iNOA
MYD_OA
SRT_iNOA
SRT_OA
UND_iNOA
UND_OA
Cell type
-log10(p.value)
3' -UTR-mediated translational regulation R-HSA-157279
Activation of the mRNA upon binding of the cap-binding complex and eIFs, and subsequent binding to 43S R-HSA-72662
Cap-dependent Translation Initiation R-HSA-72737
cellular macromolecule biosynthetic process (GO:0034645)
cellular protein metabolic process (GO:0044267)
cotranslational protein targeting to membrane (GO:0006613)
cytoplasmic translation (GO:0002181)
Disease R-HSA-1643685
Eukaryotic Translation Elongation R-HSA-156842
Eukaryotic Translation Initiation R-HSA-72613
Eukaryotic Translation Termination R-HSA-72764
flagellated sperm motility (GO:0030317)
Formation of a pool of free 40S subunits R-HSA-72689
Formation of the ternary complex, and subsequently, the 43S complex R-HSA-72695
gene expression (GO:0010467)
Gene Expression R-HSA-74160
GTP hydrolysis and joining of the 60S ribosomal subunit R-HSA-72706
Infectious disease R-HSA-5663205
Influenza Infection R-HSA-168254
Influenza Life Cycle R-HSA-168255
Influenza Viral RNA Transcription and Replication R-HSA-168273
L13a-mediated translational silencing of Ceruloplasmin expression R-HSA-156827
Major pathway of rRNA processing in the nucleolus R-HSA-6791226
Metabolism of amino acids and derivatives R-HSA-71291
Metabolism of proteins R-HSA-392499
mitochondrial electron transport, cytochrome c to oxygen (GO:0006123)
ncRNA processing (GO:0034470)
Nonsense Mediated Decay (NMD) enhanced by the Exon Junction Complex (EJC) R-HSA-975957
Nonsense Mediated Decay (NMD) independent of the Exon Junction Complex (EJC) R-HSA-975956
Nonsense-Mediated Decay (NMD) R-HSA-927802
nuclear-transcribed mRNA catabolic process (GO:0000956)
nuclear-transcribed mRNA catabolic process, nonsense-mediated decay (GO:0000184)
peptide biosynthetic process (GO:0043043)
Peptide chain elongation R-HSA-156902
protein targeting to ER (GO:0045047)
ribonucleoprotein complex assembly (GO:0022618)
Ribosomal scanning and start codon recognition R-HSA-72702
ribosomal small subunit assembly (GO:0000028)
ribosomal small subunit biogenesis (GO:0042274)
ribosome assembly (GO:0042255)
ribosome biogenesis (GO:0042254)
rRNA metabolic process (GO:0016072)
rRNA processing (GO:0006364)
rRNA processing R-HSA-72312
Selenoamino acid metabolism R-HSA-2408522
Selenocysteine synthesis R-HSA-2408557
SRP-dependent cotranslational protein targeting to membrane (GO:0006614)
SRP-dependent cotranslational protein targeting to membrane R-HSA-1799339
TP53 Regulates Metabolic Genes R-HSA-5628897
Transcriptional Regulation by TP53 R-HSA-3700989
translation (GO:0006412)
Translation initiation complex formation R-HSA-72649
Translation R-HSA-72766
viral gene expression (GO:0019080)
Viral mRNA Translation R-HSA-192823
viral process (GO:0016032)
viral transcription (GO:0019083)
Pathway
Average Expression
Percent Expressed
cellType
LEY
MYD

**Fig. S6. Percentage of total reads mapping to ribosomal protein genes in the testes with iGCAand the control groups, and single-cell RNA seq analysis of iNOA vs OA testes. a** Percentage of ribosomal genes expressed in each cell cluster of i) 3 controls from Guo et. al, ii) 2 controls from Sohni et al. iii) 1 testis with obstructive azoospermia and iv) 3 testes with iNOA. RP; Ribosomal Proteins. **b** Single cells dissociated from testis of 3 iNOA and 1 OA men were analyzed. Up **c, d** and downregulated **e** pathways between the two cohorts of patients were analyzed only in LEY and MYD populations only because these are the most represented somatic cells in the OA testis.

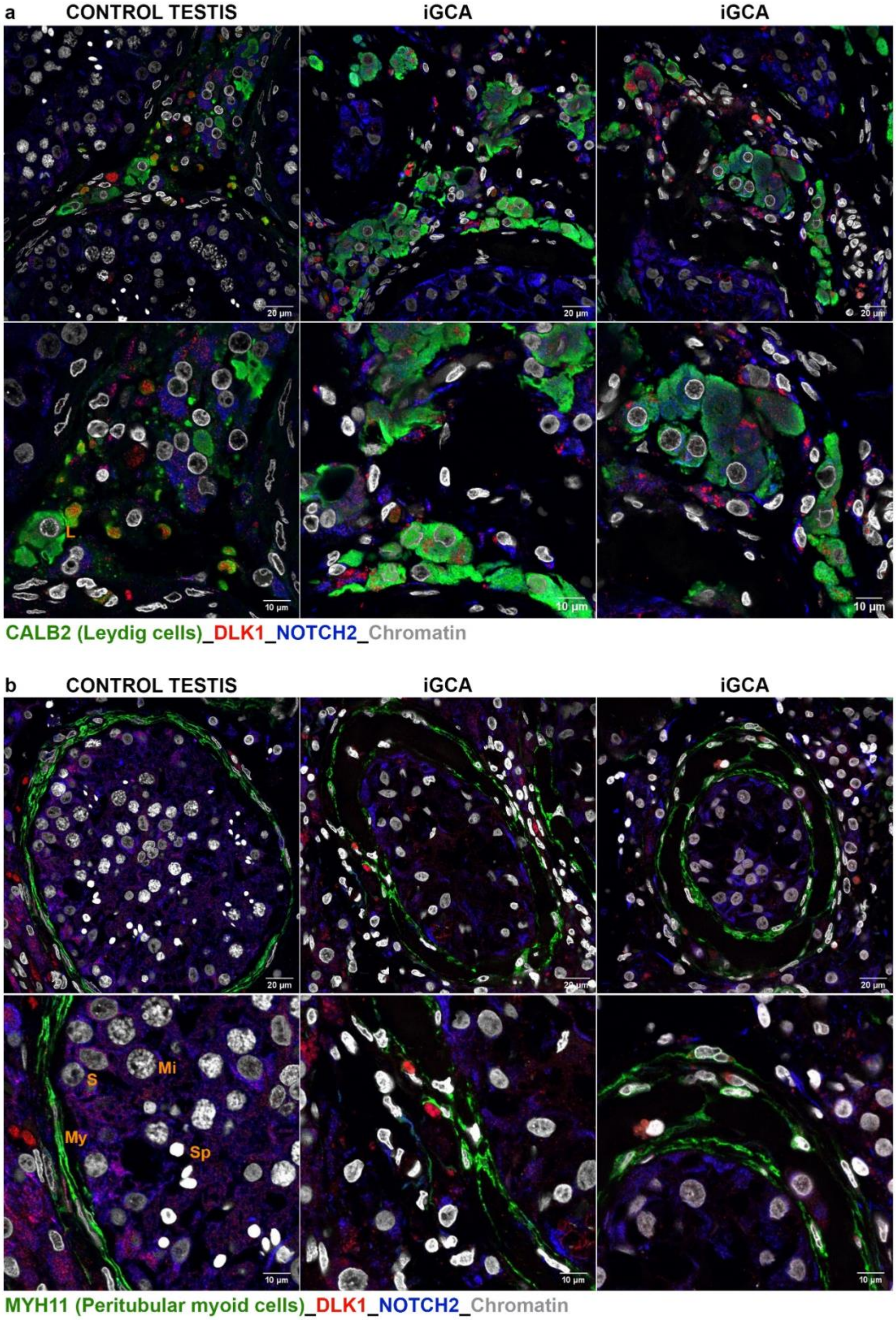


**Fig. S7. DLK1 overexpression by Leydig and peritubular myoid cells in iGCA testis.** Low and high magnification of testis parenchyma from one control testis representative of two with

similar results and iGCA testis from 2 independent donors, showing the upregulation of DLK1 in Leydig **a** and peritubular myoid cells **b** in the iGCA testis. Leydig cells were recognized through CALB2+ expression and by the chromatin condensed at the nuclear periphery. The peritubular myoid cells stained positive for MYH11 expression in the iGCA testis, the bilayer of myoid cells is separated by the hyalinized basal membrane. Sertoli cells were recognized through the presence of a characteristic DAPI negative condensed nucleolus. My; peritubular myoid cell. S; Sertoli cell. Mi; mitotic cell. Sp; sperm cell. Source data are provided with this paper.

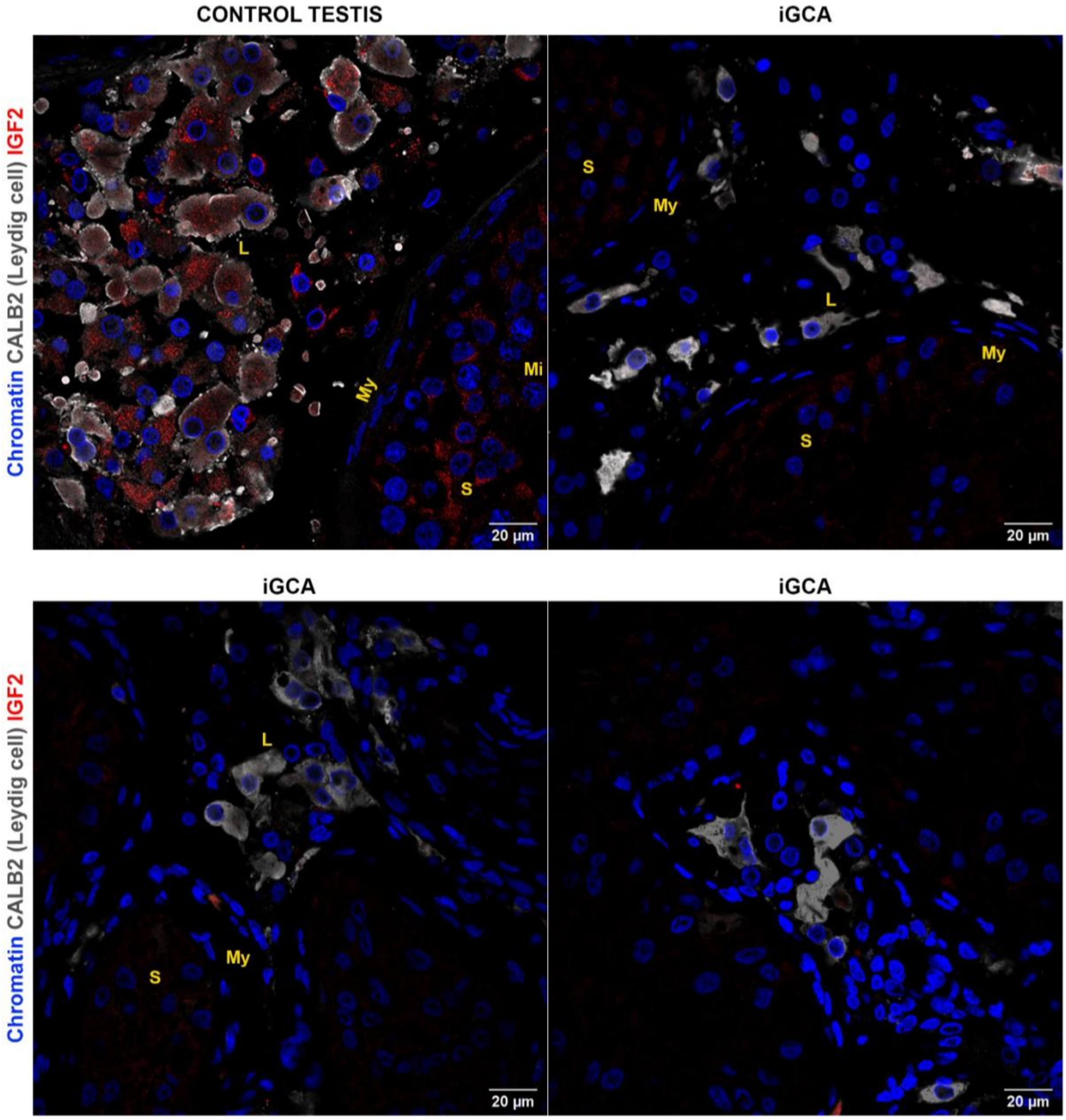


**Fig. S8. IGF2 expression by Leydig cells in CTRL and iGCA human testes.** Control testis with mitotic cells in the seminiferous tubule from 1 donor representative of two with similar results, and 3 testis parenchyma characterized by iGCA (from 3 independent donors out of 4 tested with similar results) were analysed for the expression of IGF2 in CALB2+ Leydig cells, which were also recognized through the chromatin condensed at the nuclear periphery. Sertoli cells were recognized through the presence of a characteristic DAPI negative condensed nucleolus. Double layer of peritubular myoid cells was observed in the seminiferous tubules of

iGCA testis. IGF2 expression was downmodulated in the Leydig and Sertoli cells of iGCA vs control testis, as reported in the SC-RNA seq analysis. My; peritubular myoid cell. S; Sertoli cell. Mi; mitotic cell. Source data are provided with this paper.

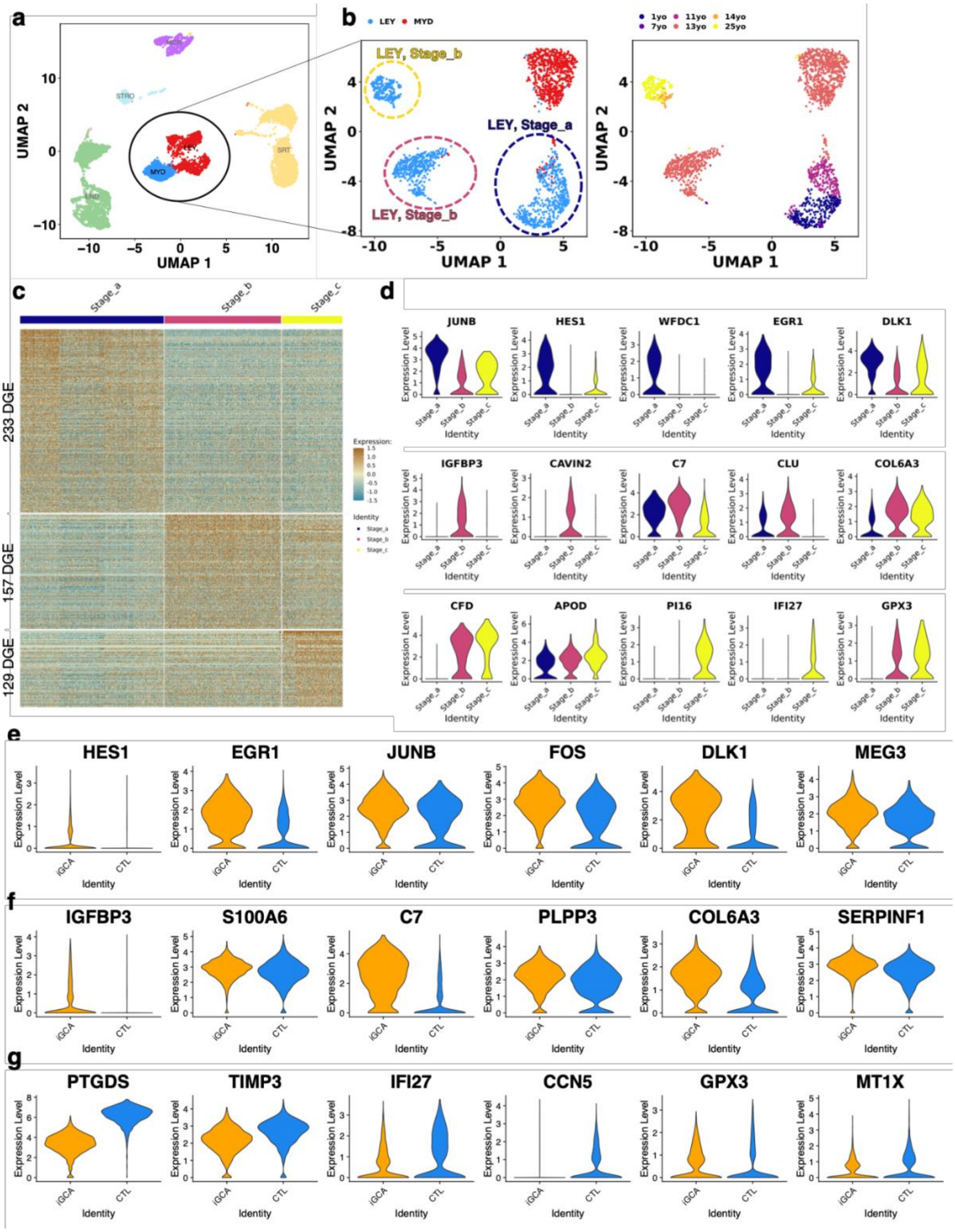


**Fig. S9. Development stage of Leydig cells.** **a** UMAP plot representing the analysis of testis cells from tissue samples of 1 neonatal testis (1yo, GSE120506), 4 prepubertal testes (7-14yo, GSE134144), 1 adult testis (25yo, GSE112013). **b** UMAP plot of re-analysis of LEY and MYD cells group by cell-type (left) and age (right). The three developmental stages of LEY cells are

highlighted in the left panel. **c** Heatmap showing the DEGs of each stage of Leydig cell maturation. DGE counts are shown on the left. **d** Violin plot showing the expression levels of the top DEGs at each stage. **e, f, g** Violin plot of the expression levels of stage-specific marker genes in iGCA LEY cells and healthy donors (from Guo et al and Sohni et al). From top to bottom: Stage A **e**, Stage B **f**, Stage C **g**.

a Paternally imprinted genes

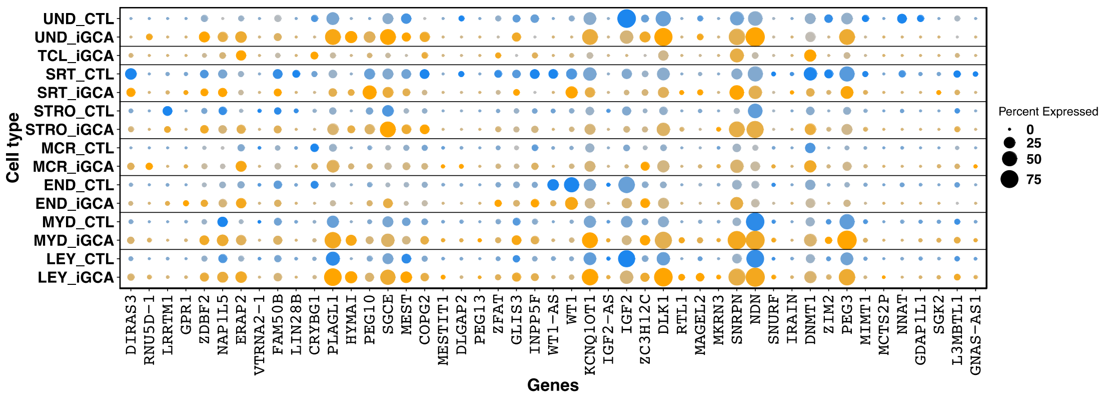


b Maternally imprinted genes

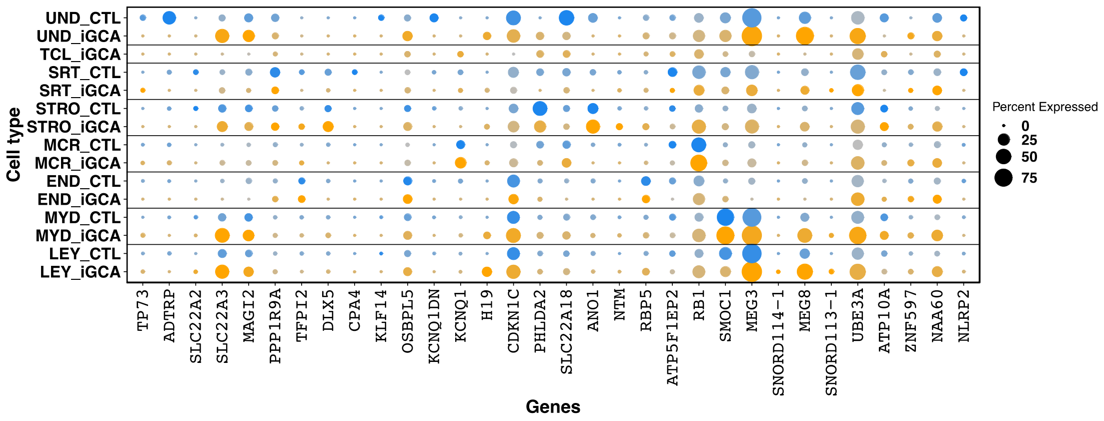


c Reclassification of immune cells from healthy testis

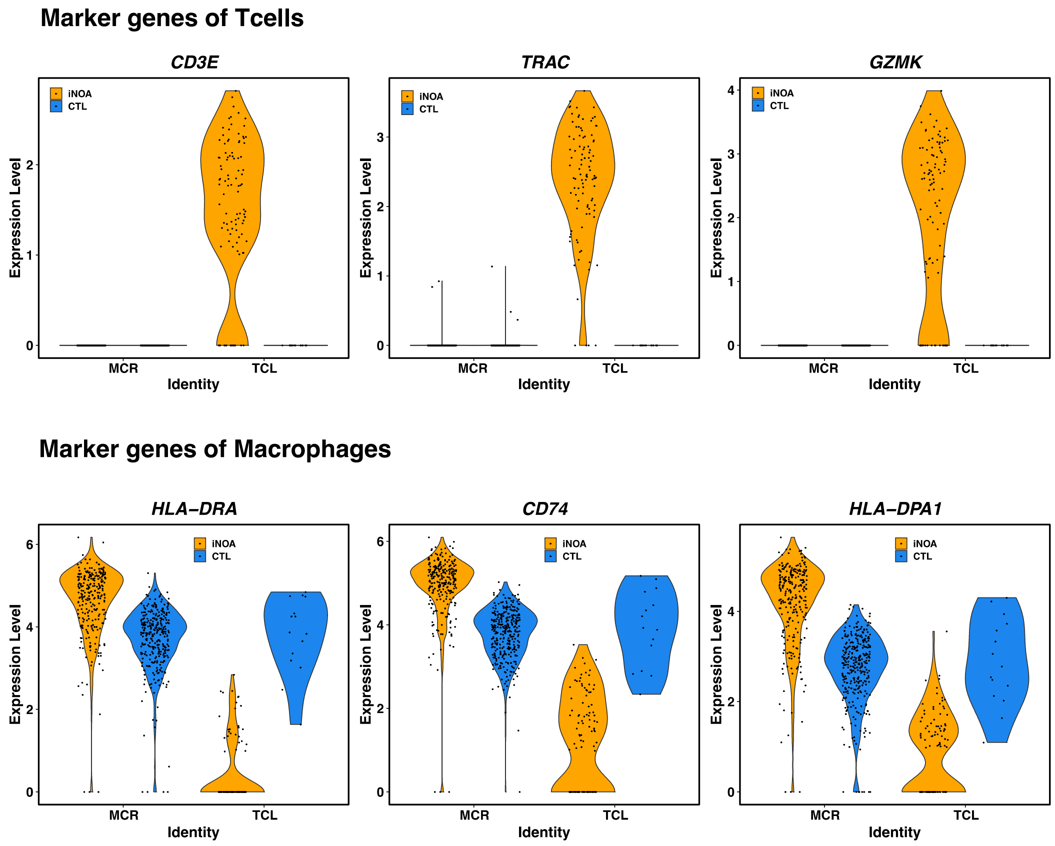

**Fig. S10. Parentally imprinted genes, and classification of immune cells from healthy donors.** Dot plot of **a** maternally and **b** paternally imprinted genes in 7 somatic cell populations from iNOA and CTL men from Guo et al and Sohni et al. **c** T cells identified in the healthy testes by Guo et al. and Sohni et al. were assigned based on the expression level of T cells marker genes (*CD3E, TRAC, GZMK*) and macrophages marker genes (*HLA-DRA, CD74, HLA-DPA1*).

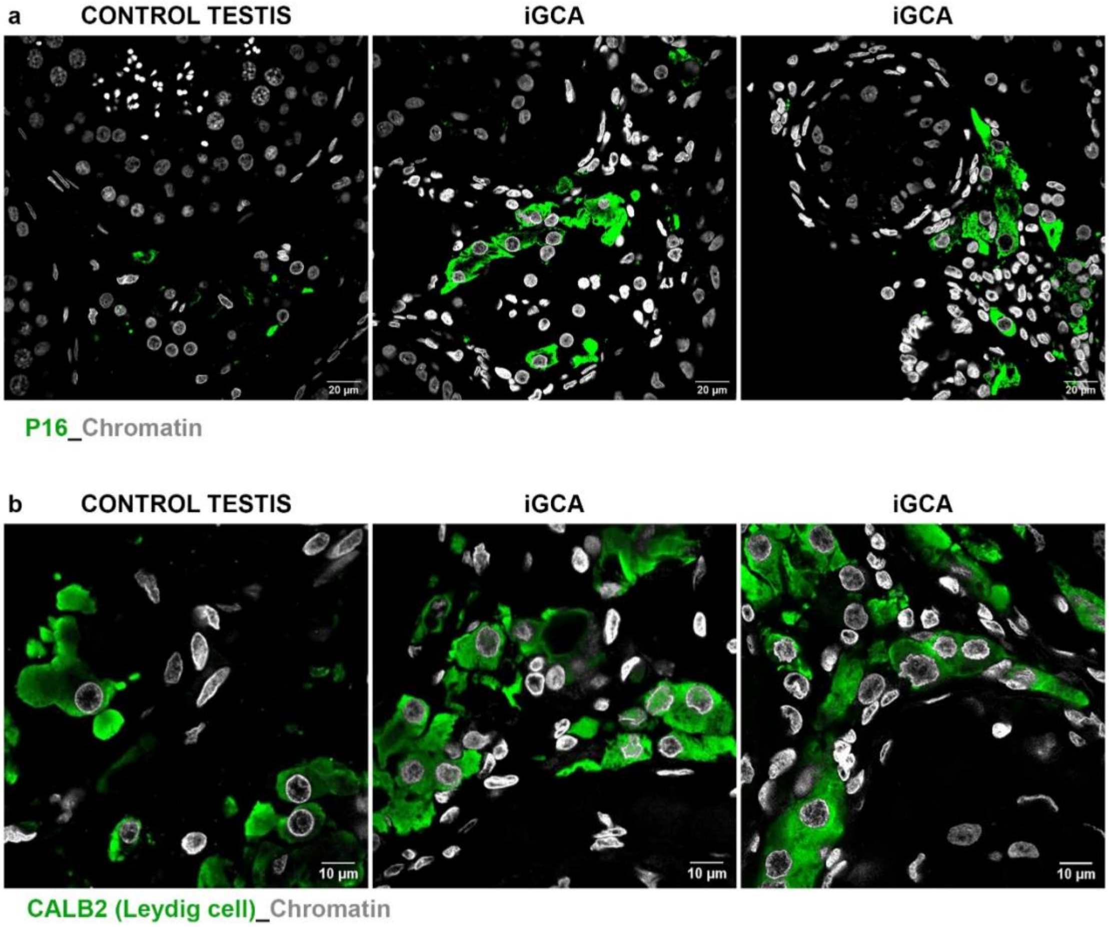


**Fig. S11. Senescence and misshapen nuclei of Leydig cells in iGCA testes. a** Overexpression of the senescence marker p16 (p16INK4a /CDKN2A) in Leydig cells in two independent iGCA specimens out of 4 tested with similar results vs CTL testis (1 donor shown out of 2 tested with similar results). **b** CALB2 staining and chromatin condensed at the nuclear periphery were used to identify Leydig cells to characterize the degree of nuclear distorsion (1 representative CTL testis out of 2 independent donors is shown, with similar results; 2 representative iGCA testis out of 4 independent donors are shown, with similar results). Source data are provided with this paper.

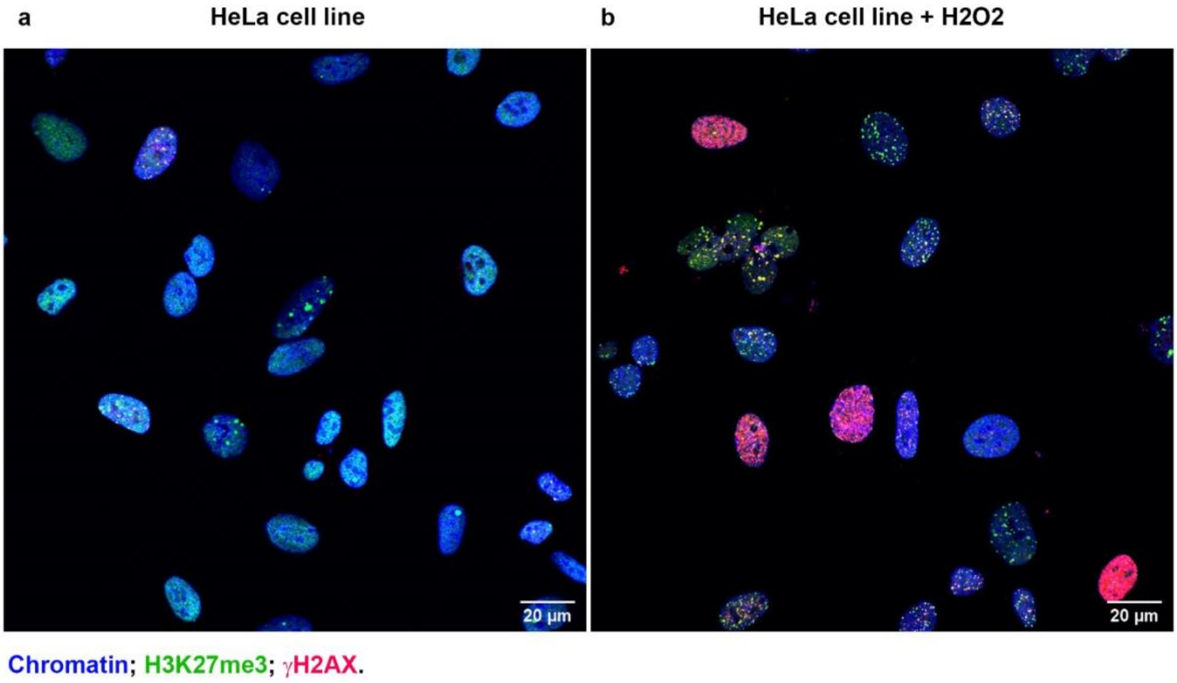


**Fig. S12. γH2AX nuclear staining after treatment with $H_2O_2$.** HeLa cells left untreated **a** or treated for 30 min with 100 µM $H_2O_2$ **b** were assayed for DNA damage by staining with the gammaH2AX antibody (red). The histone H3K27me3 Ab (green) was used as internal control. One representative experiment out of 3 independent experiments is shown, with similar results. Source data are provided with this paper.

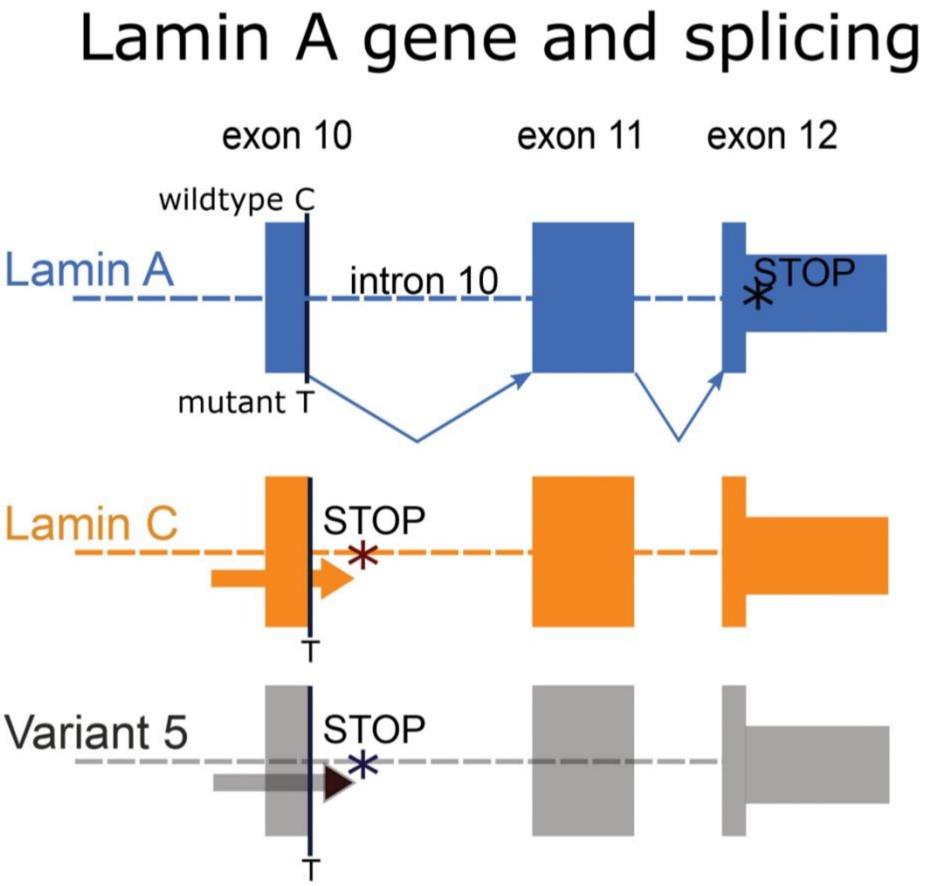


<table>
<tr><th>Column1</th><th>Column2</th><th>Column3</th><th>Column4</th></tr>
<tr><td></td><td>RefSeqs of Annotated Genomes: Homo sapiens Updated Annotation Release 109.20210226; https://www.ncbi.nlm.nih.gov/gene/4000</td><td></td><td>Ensembl website; http://www.ensembl.org/Homo_sapiens/Gene/Summary?db=core;g=ENSG00000160789;r=1:156082573-156140081</td></tr>
<tr><td>transcript variant 1/ isoform A</td><td>NM_170707.4</td><td>NP_733821.1</td><td>ENST00000368300.9|ENSG00000160789.23|OTTHUMG00000013961.6|OTTHUMT00000039200.3|LMNA-205|LMNA|3178|protein_coding|</td></tr>
<tr><td>transcript variant 2/ isoform C</td><td>NM_005572.4</td><td>NP_005563.1</td><td>ENST00000677389.1|ENSG00000160789.23|OTTHUMG00000013961.6|-|LMNA-238|LMNA|2029|protein_coding|</td></tr>
<tr><td>transcript variant 4/ isoform D</td><td>NM_001257374.3</td><td>NP_001244303.1</td><td>ENST00000448611.6|ENSG00000160789.23|OTTHUMG00000013961.6|OTTHUMT00000359799.1|LMNA-207|LMNA|1943|protein_coding|</td></tr>
<tr><td>transcript variant 5/ isoform E</td><td>NM_001282624.2</td><td>NP_001269553.1</td><td>ENST00000473598.6|ENSG00000160789.23|OTTHUMG00000013961.6|OTTHUMT00000039198.3|LMNA-212|LMNA|2015|protein_coding|</td></tr>
<tr><td>LMNA-216</td><td></td><td></td><td>ENST00000498722.2|ENSG00000160789.23|OTTHUMG00000013961.6|OTTHUMT00000359801.1|LMNA-216|LMNA|1348|retained_intron|</td></tr>
<tr><td>LMNA-229</td><td></td><td></td><td>ENST00000675667.1|ENSG00000160789.23|OTTHUMG00000013961.6|-|LMNA-229|LMNA|2826|protein_coding|</td></tr>
</table>

**Fig. S13. Scheme of *LMNA* splicing.** The cartoon summarises the splicing events producing *LaminA*, *LaminC* and *Variant 5* transcripts (blue, orange and grey, respectively) from the *LMNA* premRNA. Rectangles are the exons (exons 1 to 9 are omitted for sake of resolution). Dashed lines are the introns. The C to T transition at c.1698 (rs.4641) is indicated as a black vertical bar. The translation stop codon (asterisk) in intron 10 is used in the *LaminC* and *Variant 5* transcripts, but it is spliced out in the main transcript of *LaminA*, which exploits the stop

codon in ex 12 to terminate translation. The thickness of the arrows is proportional to the odds of having the ex10-to-ex11 splicing event as opposed to the run-through of intron 10 when T replaces the C in the ex10 Splicing Donor site. The polymorphism in the *LMNA* gene (rs4641; 1698 C>T; rs4641; His566His) was first detected by Vigoroux et al.[51]

The table reports the RefSeq and Ensembl! annotation IDs for the *Lamin A/C* spliced isoforms cited in the paper. For global info on Lamin A/C and laminopathies:

https://www.ncbi.nlm.nih.gov/gene/4000

https://www.ensembl.org/Homo_sapiens/Gene/Summary?db=core;g=ENSG00000160789;r=1:156082573-156140081;t=ENST00000498722;

https://www.ensembl.org/Homo_sapiens/Gene/Summary?db=core;g=ENSG00000160789;r=1:156082573-156140081;t=ENST00000498722.
