# Supplementary material for: Aging, inflammation and DNA damage in the somatic testicular niche with idiopathic germ cell aplasia
Source: Nat Commun. 2021 Sep 1;12:5205. doi: 10.1038/s41467-021-25544-0 (PMC8410861; doi:10.1038/s41467-021-25544-0)
Supplement: Supplementary file 3 — Description of Additional Supplementary Files [file 41467_2021_25544_MOESM3_ESM.docx]

Description of Additional Supplementary Files

Title: Supplementary Dataset 1.

Description: Marker genes of iNOA and OA as generated from Seurat Find All Markers function.

Title: Supplementary Dataset 2.

Description: Marker genes for testis somatic cell identifications.

Title: Supplementary Dataset 3.

Description: EnrichR marker genes iNOA, cluster LEY.

Title: Supplementary Dataset 4.

Description: EnrichR marker genes iNOA, cluster MYD.

Title: Supplementary Dataset 5.

Description: EnrichR marker genes iNOA, cluster SRT.

Title: Supplementary Dataset 6.

Description: EnrichR marker genes iNOA, cluster MCR.

Title: Supplementary Dataset 7.

Description: EnrichR marker genes iNOA, cluster TCL.

Title: Supplementary Dataset 8.

Description: EnrichR marker genes iNOA, cluster END.

Title: Supplementary Dataset 9.

Description: EnrichR marker genes iNOA, cluster STRO.

Title: Supplementary Dataset 10.

Description: EnrichR marker genes iNOA, cluster UND.

Title: Supplementary Dataset 11.

Description: Marker genes from reanalysis of Samples Guo et al as generated from Seurat Find All Markers function.

Title: Supplementary Dataset 12.

Description: Marker genes of the integrated object (iNOA + Guo et al and Sohni et al) as generated from Seurat Find All Markers function.

Title: Supplementary Dataset 13.

Description: Upregulated pathways from EnrichR DGE, cluster MYD.

Title: Supplementary Dataset 14.

Description: Upregulated pathways from EnrichR DGE, cluster LEY.

Title: Supplementary Dataset 15.

Description: Upregulated pathways from EnrichR DGE, cluster STRO.

Title: Supplementary Dataset 16.

Description: Upregulated pathways from EnrichR DGE, cluster SRT.

- Title: Supplementary Dataset 17.

Description: Upregulated pathways from EnrichR DGE, cluster MCR.

Title: Supplementary Dataset 18.

Description: Upregulated pathways from EnrichR DGE, cluster END.

Title: Supplementary Dataset 19.

Description: Upregulated pathways from EnrichR DGE, cluster UND.

Title: Supplementary Dataset 20.

Description: Cell-cell interactions in iGCA and CTL samples.

Title: Supplementary Dataset 21.

Description: Downregulated pathways from EnrichR DGE, cluster MYD.

Title: Supplementary Dataset 22.

Description: Downregulated pathways from EnrichR DGE, cluster LEY.

Title: Supplementary Dataset 23.

Description: Downregulated pathways from EnrichR DGE, cluster STRO.

Title: Supplementary Dataset 24.

Description: Downregulated pathways from EnrichR DGE, cluster SRT.

Title: Supplementary Dataset 25.

Description: Downregulated pathways from EnrichR DGE, cluster MCR.

Title: Supplementary Dataset 26.

Description: Downregulated pathways from EnrichR DGE, cluster END.

Title: Supplementary Dataset 27.

Description: Downregulated pathways from EnrichR DGE, cluster UND.

Title: Supplementary Dataset 28.

Description: Downregulated Pathways tabulated for cell type intersections - as in Venn Diagram Fig. 5.

Title: Supplementary Dataset 29.

Description: DGE in iNOA vs. CTRL.

Title: Supplementary Dataset 30.

Description: Marker genes of LEY developmental stages A, B and C.

Title: Supplementary Dataset 31.

Description: DGE imprinted genes.

Title: Supplementary Dataset 32.

Description: Core and patient specific imprinted genes.

Title: Supplementary Dataset 33.

Description: Celltypes_iNOA.

Title: Supplementary Dataset 34.

Description: Data accession and Samples information.

Title: Supplementary Dataset 35.

Description: LMN isoforms.

Title: Supplementary Dataset 36.

Description: List of antibody IF.
