# Supplementary material for: Aging, inflammation and DNA damage in the somatic testicular niche with idiopathic germ cell aplasia
Source: Nat Commun. 2021 Sep 1;12:5205. doi: 10.1038/s41467-021-25544-0 (PMC8410861; doi:10.1038/s41467-021-25544-0)
Supplement: Supplementary file 35 — Supplementary Dataset 32 [file 41467_2021_25544_MOESM35_ESM.docx]

**Table Supplementary 32. CORE of imprinted genes in all 3 iGCA testes.**

| **Gene** | **iGCA#1** | **iGCA#2** | **iGCA#3** | **OA** | **W1** | **W2** | **Guo1** | **Guo2** | **Guo3** |
| --- | --- | --- | --- | --- | --- | --- | --- | --- | --- |
| IGF2 (p) | Bi | Bi | Bi | No SNPs | No SNPs | No SNPs | No SNPs | No SNPs | No SNPs |
| DLK1 (p) | Bi | Bi | Bi | No SNPs | No SNPs | No SNPs | No SNPs | No SNPs | No SNPs |
| HYMAI (p) | Bi | Bi | Bi | ND | ND | ND | ND | ND | ND |
| ERAP2 (p) | Bi | Bi | Bi | ND | ND | ND | ND | ND | ND |
| PEG3 (p) | No SNPs | No SNPs | No SNPs | No SNPs | No SNPs | No SNPs | No SNPs | No SNPs | No SNPs |
| H19 (m) | No SNPs | No SNPs | No SNPs | No SNPs | No SNPs | No SNPs | ND | ND | ND |
| MEG8 (m) | No SNPS | Bi | Bi | ND | ND | ND | ND | ND | ND |

Detection of imprinted genes for which altered expression was found in the somatic cells of testis with iGCA vs control testis reported in the Wilkinson and Guo datasets. iGCA; idiopathic germ cell aplasia. OA; obstructive azoospermia. W1-W2; control testis from the Wilkinson dataset. Guo1-Guo3; control testis from the Guo dataset. No SNPs; absence of single nucleotide polymorphisms (SNPs) associated to disease. ND; not defined, because poor expression or no expression. Bi; bi-allelic expression. (m); maternal gene expression. (p); paternal gene expression.

**Table Supplementary 33. Imprinted genes specific for each patient with iGCA.**

|  | **iGCA#1** | **iGCA#2** | **iGCA#3** |
| --- | --- | --- | --- |
| **Patient specific imprinted genes** | UBE3A (m) |  |  |
|  | SLC22A3 (m) |  |  |
|  | MEG3 (m) |  | MEG3 (m) |
|  |  | MAGI2 (m) |  |
|  | PEG10 (p) |  |  |
|  | ZDBF2 (p) |  |  |
|  |  | RTL1 (p) | RTL1 (p) |
|  |  | COPG2 (p) |  |
|  |  | NAP1L5 (p) |  |
|  |  |  | DIRAS3 (p) |

Imprinted genes for which altered expression was found in the somatic cells of testis with iGCA vs control testis reported in the Wilkinson and Guo datasets. (m); maternal gene expression. (p); paternal gene expression.
