# Supplementary material for: Aging, inflammation and DNA damage in the somatic testicular niche with idiopathic germ cell aplasia
Source: Nat Commun. 2021 Sep 1;12:5205. doi: 10.1038/s41467-021-25544-0 (PMC8410861; doi:10.1038/s41467-021-25544-0)
Supplement: Supplementary file 38 — Supplementary Dataset 35 [file 41467_2021_25544_MOESM38_ESM.docx]

**Table S3. List of primary and secondary antibodies used for immunofluorescence staining.**

| **Antibody** | **Code** | **Company** | **Host** | **Antibody concentration** | **Dilution** | **Antigen retrieval** |
| --- | --- | --- | --- | --- | --- | --- |
| INSL3 | NBP1-81223 | Novus Biological | rabbit | 0,1mg/ml | 1:2000 | Citrate |
| CALB2 | HPA007305 | Merck Life Science | rabbit | 1,1mg/ml | 1:1000 | EDTA/citrate |
| MYH11 | HPA015310 | Merck Life Science | rabbit | 0,1mg/ml | 1:100 | Citrate |
| MYH11 | HPA015310 | Merck Life Science | rabbit | 0,1mg/ml | 1:200 | EDTA |
| IGF2 | MA517096 | Thermo Fisher Scientific | mouse | 1mg/ml | 1:200 | Citrate |
| NOTCH2 | MA524274 | Thermo Fisher Scientific | rat | 0,5mg/ml | 1:100 | EDTA |
| DLK-1 | ab119930 | Abcam | mouse | 1mg/ml | 1:100 | EDTA |
| p16INK4a | LS-B1347-50 | LSBio | rabbit | 1mg/ml | 1:100 | EDTA |
| H2AXS139P | 05-636 | Merck Life Science | mouse | 1mg/ml | 1:500 | Citrate |
| **H3k27me3** | **07-449** | Sigma Aldrich | rabbit | 1 mg/ml | 1:500 | Citrate |

| **Secondary Antibody** | **Fluorocrome** | **Code** | **Company** | **Antibody concentration** | **Dilution** |
| --- | --- | --- | --- | --- | --- |
| Donkey anti-Rabbit IgG (H+L) | Alexa Fluor 488 | A32790 | Thermo Fisher Scientific | 2mg/ml | 1:2000 |
| Donkey anti-Mouse IgG (H+L) | Alexa Fluor 555 | A32773 | Thermo Fisher Scientific | 2mg/ml | 1:2000 |
| Donkey Anti-Rat IgG (H+L) | Alexa Fluor 647 | ab150155 | Abcam | 2mg/ml | 1:1000 |
| NucBlue Live ReadyProbe Reagent | Hoechst 33342 | R37605 | Thermo Fisher Scientific |  | 1:10 |
