# Supplementary material for: Aging, inflammation and DNA damage in the somatic testicular niche with idiopathic germ cell aplasia
Source: Nat Commun. 2021 Sep 1;12:5205. doi: 10.1038/s41467-021-25544-0 (PMC8410861; doi:10.1038/s41467-021-25544-0)
Supplement: Supplementary file 40 — Reporting Summary [file 41467_2021_25544_MOESM40_ESM.pdf]

nature research

Corresponding author(s): Alfano Massimo

Last updated by author(s): Aug 4, 2021

# Reporting Summary

Nature Research wishes to improve the reproducibility of the work that we publish. This form provides structure for consistency and transparency in reporting. For further information on Nature Research policies, see our Editorial Policies and the Editorial Policy Checklist.

## Statistics

For all statistical analyses, confirm that the following items are present in the figure legend, table legend, main text, or Methods section.

| n/a | Confirmed | |
|---|---|---|
| ☐ | ☒ | The exact sample size (*n*) for each experimental group/condition, given as a discrete number and unit of measurement |
| ☐ | ☒ | A statement on whether measurements were taken from distinct samples or whether the same sample was measured repeatedly |
| ☐ | ☒ | The statistical test(s) used AND whether they are one- or two-sided<br>*Only common tests should be described solely by name; describe more complex techniques in the Methods section.* |
| ☐ | ☒ | A description of all covariates tested |
| ☐ | ☒ | A description of any assumptions or corrections, such as tests of normality and adjustment for multiple comparisons |
| ☐ | ☒ | A full description of the statistical parameters including central tendency (e.g. means) or other basic estimates (e.g. regression coefficient) AND variation (e.g. standard deviation) or associated estimates of uncertainty (e.g. confidence intervals) |
| ☐ | ☒ | For null hypothesis testing, the test statistic (e.g. *F*, *t*, *r*) with confidence intervals, effect sizes, degrees of freedom and *P* value noted<br>*Give P values as exact values whenever suitable.* |
| ☒ | ☐ | For Bayesian analysis, information on the choice of priors and Markov chain Monte Carlo settings |
| ☐ | ☒ | For hierarchical and complex designs, identification of the appropriate level for tests and full reporting of outcomes |
| ☒ | ☐ | Estimates of effect sizes (e.g. Cohen's *d*, Pearson's *r*), indicating how they were calculated |

*Our web collection on statistics for biologists contains articles on many of the points above.*

## Software and code

Policy information about availability of computer code

| | |
|---|---|
| Data collection | - Cell Ranger v.3.0.2 (10x Genomics) was used for demultiplex sequencing data (raw base call - BCL) with cellranger mkfastq;<br>- UMI_tools whitelist was used to identify the number of cell barcodes to use in downstream analysis with the --set-cell-number option (umi_tools v1.0.0);<br>- UMI_tools extract was used to extract the selected cell barcodes (option --filter-cell-barcode, umi_tools v1.0.0);<br>- Extracted reads were aligned with STAR aligner (v2.5.3a) to the primary assembly of human genome (GRCh38) with parameter '--outFilterMultimapNmax 1' (to exclude multi-mapping reads);<br>- Mapped reads were assigned to gene_name attributes from GENCODE basic annotation for GRCh38 (version 31) using Subread featureCounts (v1.6.4). Results were save in BAM files with option 'featureCounts -R BAM';<br>- the BAM files were sorted and indexed with samtools (v1.9).<br>- UMI_tools count was used to summarize gene counts for each cell (umi_tools count --per-gene --gene-tag=XT --assigned-status-tag=XS --per-cell'), umi_tools v1.0.0. |
| Data analysis | - Cell type identification and clustering analysis was performed with the Seurat program (v.3.1.5), within the R environment (v3.6.1);<br>- Differential Gene Expression analysis in each cell cluster was performed with the FindMarkers function of Seurat (v.3.1.5), using default parameters (wilcox test, logfc.threshold = 0.25 and min.pct = 0.1);<br>- Enrichment analysis was performed with the EnrichR package (v.2.1), which provides an R interface to the 'Enrichr' databases and statistics;<br>- Jaccard indices were evaluated with the CRAN package philentropy (Version 0.5.0), and used to compute an adjacency matrix for all possible pairs of significant pathways, which was then represented with the CRAN package pheatmap (Version 1.0.12);<br>- For LMNA differential isoform quantification analysis was performed by kallisto pseudo-alignment tool, using Gencode v37 basic annotation transcript fasta as annotation;<br>- CellPhoneDB v.2.0 was used to quantify the number of total interactions between cell types, using the statistical_analysis method, performing 100 iterations for the statistical analysis. |

-Imaging analysis was performed with the software ImageJ version 1.5.
-The ImageJ plug-in to quantify fibrillar structures in raw microscopy images was, FibrilTool, was downloaded from the manuscript with DOI: 10.1038/nprot.2014.024

For manuscripts utilizing custom algorithms or software that are central to the research but not yet described in published literature, software must be made available to editors and reviewers. We strongly encourage code deposition in a community repository (e.g. GitHub). See the Nature Research guidelines for submitting code & software for further information.

## Data

Policy information about availability of data

All manuscripts must include a data availability statement. This statement should provide the following information, where applicable:

- Accession codes, unique identifiers, or web links for publicly available datasets
- A list of figures that have associated raw data
- A description of any restrictions on data availability

The single cell RNA sequencing data from 1 testing with obstructive azoospermia and 3 testis with iGCA from this study were deposited in GEO : accession code GSE154535.

The testicular cells scRNA-seq data from human adult with normal spermatogenesis are publicly available from the studies of Guo et al., Cell Research, 2018 (GEO: GSE112013) and Sohni et al., Cell Reports, 2019 (GEO:GSE124263).
The testicular cells scRNA-seq data from neonatal, pre-pubertal and adult testis are publicly available from the studies of Guo et al., Cell Research, 2018 (GEO: GSE112013; GEO:GSE120506), Sohni et al., Cell Reports, 2019 (GEO:GSE124263) and Guo et al., Cell Stem Cell, 2020 (GEO:GSE134144).

Source data for all main figures are provided with this paper.

No restriction

# Field-specific reporting

Please select the one below that is the best fit for your research. If you are not sure, read the appropriate sections before making your selection.

☒ Life sciences ☐ Behavioural & social sciences ☐ Ecological, evolutionary & environmental sciences

For a reference copy of the document with all sections, see nature.com/documents/nr-reporting-summary-flat.pdf

# Life sciences study design

All studies must disclose on these points even when the disclosure is negative.

| | |
|---|---|
| Sample size | Sample size was based on the availability of biospecimens, such as fresh tissue for sc-RNAseq, formalin-fixed paraffin embedded tissue and frozen serum for validation of the information obtained through sequencing. Biospecimens included solid and liquid tissues from iGCA testis, non-neoplastic testis with normal spermatogenesis, peripheral serum from patients with iGCA and healthy controls with proven fertility.<br><br>Fresh testes parenchyma, peripheral blood, clinical and anamnestic data from 3 men with iGCA men (age = 32, 37, 41 y) and 1 man with obstructive azoospermia (age = 37 y), for Sc-RNAseq (Authorization Protocol Infertilità-2015, amended on March 2016).<br><br>Formalin-fixed paraffin embedded testes parenchyma with iGCA (n=10) and idiopathic hypospermatogenesis (n=10), peripheral blood, clinical and anamnestic data from 20 iNOA men (median age=37 y, interquartile range=32-38 y); the non-neoplastic tissue of 5 men submitted to unilateral orchiectomy for non-metastatic seminoma (median age=41 y, interquartile range=38-45 y). These tissues were used for immunohistochemical analysis of collagen I and collagen IV. Authorization Protocol Infertilità-2015, amended on March 2016.<br><br>Peripheral blood, clinical and anamnestic data from a cohort of 44 iNOA men with germ cell aplasia (median age=37 y, interquartile range=33-39 y), for hormonal profile (Authorization Protocol Infertilità-2015, amended on March 2016).<br><br>Peripheral blood, clinical and anamnestic data from a cohort of 102 men with proven fertility, and classified upon sperm analysis (median age=37 y, interquartile range=33-40 y), for hormonal profile (Authorization Protocol URIMALES-2016, amended on February 2018).<br><br>The sample sizes were sufficient to analyze meaningful differences in the available cohorts. |
| Data exclusions | No data were excluded. |
| Replication | No replication possible as study used biospecimens collected during surgical intervention, either from microTESE or orchiectomy. Human testis specimens from microTESE were unique, and the low amount of the specimen did not allow for replicate studies. |
| Randomization | Participants were allocated into the experimental group according to the result from the histological analysis of the tissue and clinical information. All human specimens were from male, age range (35-40 y), Caucasian and with the same pathology as idiopathic germ cell aplasia. The specimen from one patient with obstructive azoospermia and the same covariates was used as positive control. |
| Blinding | Sc-RNAseq data acquisition from fresh tissues were performed blinded to histological diagnosis. All other analysis were not blinded, and the |

samples selected to match the age of the participants; blinding approach does not apply to this study because one single group of disease was analyzed (idiopathic germ cell aplasia).

# Reporting for specific materials, systems and methods

We require information from authors about some types of materials, experimental systems and methods used in many studies. Here, indicate whether each material, system or method listed is relevant to your study. If you are not sure if a list item applies to your research, read the appropriate section before selecting a response.

## Materials & experimental systems

| n/a | Involved in the study | |
|---|---|---|
| ☐ | ☒ | Antibodies |
| ☒ | ☐ | Eukaryotic cell lines |
| ☒ | ☐ | Palaeontology and archaeology |
| ☒ | ☐ | Animals and other organisms |
| ☐ | ☒ | Human research participants |
| ☒ | ☐ | Clinical data |
| ☒ | ☐ | Dual use research of concern |

## Methods

| n/a | Involved in the study | |
|---|---|---|
| ☒ | ☐ | ChIP-seq |
| ☒ | ☐ | Flow cytometry |
| ☒ | ☐ | MRI-based neuroimaging |

## Antibodies

Antibodies used

Primary antibodies against human antigens and used in IHC analysis were:
- anti-Collagen I (Ab34710 from Abcam, Cambridge, UK), which has been used in more than 800 published studies (https://www.abcam.com/collagen-i-antibody-ab34710.html)
- anti-Collagen IV (M0785 from Dako, Denmark), which has been used in 8 published paper showing IHC data (https://www.citeab.com/antibodies/2414799-m0785-collagen-iv-concentrate)
- anti-HMGB1 (Abcam 18256)

The following primary Ab against human antigens used in immunofluorescence analysis, and the antigen retrieval of FFPE tissue and Ab dilutions are reported in the Supplementary Table S3;
- anti-INSL3 (NBP1-81223, Novus Biological)
- anti-CALB2 (HPA007305, Merck Life Science)
- anti- MYH11 (HPA015310, Merck Life Science)
- anti-IGF2 (MA517096, Thermo Fisher Scientific)
- anti- NOTCH2 (MA524274, Thermo Fisher Scientific)
- anti- p16INK4a (LS-B1347-50, LSBio)
- anti- H2AXS139P (05-636, Merck Life Science)
- anti- H3k27me3 (07-449, Sigma Aldrich)
- anti-gH2AXS (H2AX139P; code 05-636 Merck Life Science)

The following secondary Ab were used in immunofluorescence, and the Ab dilutions reported in the Supplementary Table S35);
- Donkey anti-Rabbit IgG (H+L) (A32790, Thermo Fisher Scientific)
- Donkey anti-Mouse IgG (H+L) (A32773, Thermo Fisher Scientific)
- Donkey Anti-Rat IgG (H+L) (ab150155, Abcam)

Validation

Negative control for immunohistochemical analsysis was obtained by omitting the primary Ab.
All primary antibodies were certified by the manufacturer; the stainings provided in our study were checked for antigen localization in Protein Atlas.

## Human research participants

Policy information about studies involving human research participants

| | |
|---|---|
| Population characteristics | Population characteristics of human research participants have been detailed in the Methods section.<br>Three participants were Caucasian men, in the range of 35-40 year, with therapy-naive idiopathic germ cell aplasia.<br>The specimen from one patient with obstructive azoospermia and the same covariates was used as positive control.<br>No additional covariate population characteristics were utilized. |
| Recruitment | Specimens were collected at one tertiary-referral center for reproductive medicine (IRCCS San Raffaele, Milan, Italy). Patients were recrutited blindly and only after histological analysis assigned to the study. |
| Ethics oversight | Data collection followed the principles outlined in the Declaration of Helsinki; all patients signed an informed consent agreeing to supply their own anonymous information and tissue specimens. The study was approved by the Institutional Review Board (Ethic Committee IRCCS Ospedale San Raffaele, Milan, Italy), and was composed by 2 protocols for collecting data and using bio-specimens from infertile (Authorization Protocol Infertilità-2015, amended on March 2016) and fertile men (Authorization Protocol URIMALES-2016, further amended on March 2018), and 1 protocol for biobanking (Authorization Protocol URI001-2010, further amended on December 2015). All methods were carried out in accordance with the approved guidelines. |

Note that full information on the approval of the study protocol must also be provided in the manuscript.
